# Integrating computational lead optimization diagnostics with analog design and candidate selection

**DOI:** 10.2144/fsoa-2019-0131

**Published:** 2020-01-24

**Authors:** Dimitar Yonchev, Jürgen Bajorath

**Affiliations:** 1 Department of Life Science Informatics, B-IT, LIMES Program Unit Chemical Biology & Medicinal Chemistry, Rheinische Friedrich-Wilhelms-Universität, Endenicher Allee 19c, D-53113 Bonn, Germany

**Keywords:** analog design, candidate prioritization, computational diagnostics, decision support, Free-Wilson analysis, lead optimization, medicinal chemistry, potency prediction

## Abstract

**Aim:**

Combining computational lead optimization diagnostics with analog design and computational approaches for assessing optimization efforts are discussed and the compound optimization monitor is introduced.

**Methods:**

Approaches for compound potency prediction are described and a new analog design algorithm is introduced. Calculation protocols are detailed.

**Results & discussion:**

The study rationale is explained. Compound optimization monitor diagnostics are combined with a thoroughly evaluated approach for compound design and candidate prioritization. The diagnostic scoring scheme is further extended.

**Future perspective:**

Opportunities for practical applications of the integrated computational methodology are described and further development perspectives are discussed.

## Lay abstract

Compound optimization is a central task in medicinal chemistry, which has many potential pitfalls. Computational approaches that help to better understand and guide chemical optimization efforts are highly desirable, but only a few are currently available. We have aimed to develop a computational methodology that combines, for the first time, the evaluation of progress in chemical optimization with the design of new candidate compounds.

## Graphical abstract

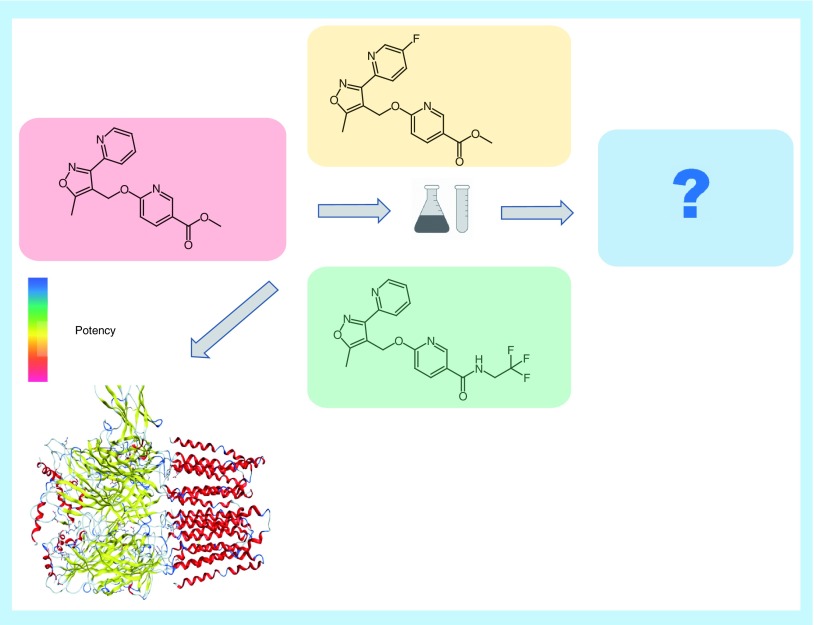

Chemical optimization efforts play a central role in the practice of medicinal chemistry [1]. During lead optimization (LO), many analogs of initially prioritized active compounds must typically be generated until candidate status is reached. However, despite large compound numbers, work on analog series (ASs) must often be terminated when required optimization criteria cannot be reached. The need to abandon large-magnitude LO efforts results in a significant loss of time and resources, which causes major problems for medicinal chemistry. Accordingly, any approaches that help to evaluate LO projects and estimate the odds of success are highly desirable. For this purpose, computational evaluation of LO is particularly attractive. However, in addition to quantitative structure–activity relationship (QSAR) approaches that are long used to predict the potency of newly designed analogs [2,3], only few computational methods are currently available that aid in planning or assessing LO efforts [4–9]. These methods include multiparameter optimization and other statistical techniques to evaluate compound property progression or identify compounds that strongly contribute to structure–activity relationships (SARs) [4–7]. None of these approaches provide comprehensive LO diagnostics or combines data analysis with molecular design. Recently, a conceptually different computational methodology has been introduced to address the questions if an AS might be chemically saturated and if further SAR progression might be expected [8,9]. The analysis makes it also possible to estimate if sufficient numbers of analogs have been generated for a given series. Hence, the evaluation of chemical saturation and SAR progression was combined to provide decision support during LO [8]. These efforts have led to the development of the compound optimization monitor (COMO) program [9]. By design, the COMO approach is diagnostic in nature, similar to two other SAR evaluation methods [6,7]. However, a special feature of COMO’s additional chemical saturation analysis component is that it utilizes populations of virtual analogs (VAs) to chart chemcial space for given ASs. These VAs are specifically generated for each AS and might thus also be evaluated as candidate compounds for synthesis. Accordingly, the COMO method might be further extended to compound design and the prediction of preferred candidates. This would provide a unique methodological combination of chemical saturation and SAR diagnostics with prospective compound design. However, achieving this goal requires the incorporation of approaches for AS-specific VA selection and candidate prediction. Herein, we report the extension of COMO to include the design and prioritization of candidate compounds for LO.

## Methods

### COMO diagnostic concept

The COMO approach, its scoring scheme and parameter optimization have been described in detail [9]. In the following, a summary of the COMO concept is presented as a basis for rationalizing its extension.

COMO evaluates chemical saturation and SAR progression of ASs by determining how extensively and densely chemical space around a given series is covered. In addition, COMO determines if significant potency variations among existing analogs (EAs) and increases in potency are observed during LO. The assessment relies on defining chemical neighborhoods (NBHs) of EAs and on using populations of VAs for given ASs to map NBHs and surrounding chemical space. VAs are currently generated using a pool of more than 32,000 unique substituents with at most 13 heavy atoms that were extracted from bioactive compounds on the basis of retrosynthetic criteria. For VA generation, the core structure of an AS is isolated while retaining substitution site information through atom indices. Then, predefined numbers of VAs are enumerated according to retrosynthetic rules using the substituent library. At individual substitution sites, an AS-specific likelihood of hydrogen substituents is taken into account [9]. All EAs and the corresponding VA population are then projected into a chemical reference space where overlapping and nonoverlapping NBHs of EAs are analyzed and their VA content is determined. Then, potency variations of EAs with overlapping and populated NBHs are quantified.

This analysis concept yields multiple scores for evaluating LO progression. COMO key scores account for chemical saturation and SAR progression. The chemical saturation score S is composed of two components quantifying the coverage and density of chemical space.

The coverage score C is defined as the proportion of VAs that populate NBHs of EAs:

(Eq. 1) $$C\;=\;\frac{n_{N}}{n_{V}}$$

Variables *n_N_* and *n_V_* refer to the number of VAs in NBHs and the total number of VAs, respectively. The C score has the range [0,1].

In addition, a term *d_mean_* is introduced as the number of overlapping NBHs containing VAs (*NBH_O_VA_*) relative to the total number of VAs falling into NBHs of EAs:

(Eq. 2) $$d_{mean}\;=\;\frac{NBH_{O\_VA}}{n_{N}}$$

The density score D with range [0,1] is then calculated as:

(Eq. 3) $$D\;=\;1-\frac{1}{d_{mean}}$$

Chemical saturation score S combines coverage and sampling density of chemical reference space and is obtained as the harmonic mean of score components C and D:

(Eq. 4) $$S\;=\;\frac{2CD}{C+D}$$

Furthermore, SAR progression is assessed by determining potency variations of EAs sharing VAs in overlapping NBHs, which provides a measure of SAR discontinuity of a given AS. For a given VA, parameter ${\bar{\Delta }}_{i}$ accounts for the potency range among *m_i_* associated analogs. It is calculated as the mean potency difference over all pairs of *m_i_* EAs. In addition, *pot_j_* and *pot_k_* represent the logarithmic potency of analog *j* and *k*, respectively:

(Eq. 5) $$\begin{array}{l} \bar{\Delta_{i}}\;=\;\frac{2}{m_{i}(m_{i}-1)}{\text{Σ}}_{\begin{array}{l} j,k=1\\ j<k \end{array}}^{m_{i}}\;\left|pot_{j}-pot_{k}\right| \end{array}$$

The SAR progression score P is then calculated as the mean over all VAs in NBHs using their ${\bar{\Delta }}_{i}$ values and a weighting scheme $w_{i}\;=\;\frac{1}{m_{i}}$ if *m_i_* >1 and *w_i_* = 0 if *m_i_* = 1:

(Eq. 6) $$P\;=\;\frac{1}{\Sigma_{i=1}^{n_{N}}\;w_{i}}\;\Sigma_{i=1}^{n_{N}}\;w_{i}\;{\bar{\Delta }}_{i}$$

The COMO calculations reported herein were carried out as described previously [9] using a seven-dimensional (7D) chemical reference space and a population of 2000 VAs per AS. In each case, S and P scores were calculated to illustrate the characterization of ASs.

### AS

For our analysis, new ASs with activity against a given target and available high-confidence potency measurements of inhibition constant (K_i_) or half maximal inhibitory (IC_50_) values were extracted from ChEMBL (version 25) [10]. The ASs were identified using a previously reported algorithm [11] following matched molecular pair (MMP) fragmentation [12,13] of bioactive compounds on the basis of retrosynthetic rules [14,15]. An MMP is defined as a pair of compounds that are only distinguished by a chemical modification at a single site [12]. Systematic fragmentation of exocyclic single bonds generates MMP cores and substituent fragments. Following fragmentation, the AS identification algorithm assembles series with a shared core and single or multiple substitution sites [11]. For our analysis, the 24 largest ASs with more than 100 compounds (max. 264) and multiple (two to six) substitution sites were considered. They contained more compounds than previously investigated ASs and were active against 16 distinct targets. Table 1 summarizes their composition.

**Table 1.** Analog series.

AS ID	Target name	ChEMBL Target ID	# Subst. Sites	# EAs	# FW EAs	# FW VAs
1	Serine/threonine-protein kinase mTOR	2842	2	153	27	264
2	Acetyl-CoA carboxylase 2	4829	2	112	0	218
3	Acetyl-CoA carboxylase 2	4829	6	149	33	3812
4	Adenosine A2b receptor	255	6	129	72	392
5	GABA receptor alpha-5 subunit	5112	2	193	118	1647
6	Purinergic receptor P2Y12	2001	2	237	145	2766
7	Vanilloid receptor	4794	3	162	0	0
8	Mitogen-activated protein kinase kinase kinase 12	1908389	2	111	44	1844
9	5-lipoxygenase activating protein	4550	3	259	162	5204
10	5-lipoxygenase activating protein	4550	2	100	4	96
11	Epidermal growth factor receptor erbB1	203	2	106	0	306
12	Sodium channel protein type IX alpha subunit	4296	3	146	40	2860
13	Acetyl-CoA carboxylase 2	4829	3	100	10	145
14	Proteinase activated receptor 4	4691	5	117	8	212
15	Acetyl-CoA carboxylase 2	4829	3	128	54	319
16	p53-binding protein Mdm-2	5023	4	149	72	381
17	Acetyl-CoA carboxylase 2	4829	3	116	81	331
18	Sodium channel protein type IX alpha subunit	4296	5	151	17	1736
19	P2X purinoceptor 3	2998	6	102	84	336
20	MAP kinase ERK2	4040	2	264	0	262
21	Tyrosine-protein kinase SYK	2599	5	173	43	5755
22	Prostaglandin E synthase	5658	3	168	63	3893
23	Tyrosine-protein kinase SYK	2599	3	168	72	1887
24	5-lipoxygenase activating protein	4550	2	126	0	124

The table summarizes the composition of ASs used herein and reports the proportion of existing analogs and newly generated virtual analogs that qualify for Free-Wilson potency prediction, as discussed in the text. ‘# Subst. Sites’ reports the number of substitution sites per AS.

AS: Analog series; EA: Existing analog; FW: Free-Wilson; ID: Identification; VA: Virtual analogs.

### Linear & ridge regression

Linear regression (LR) is the simplest and most widely used statistical approach for numerical value predictions [16]. In QSAR modeling, LR is applied assuming the presence of linear relationships between numerical chemical features and biological activity [3]. The predictive performance of LR models inevitably suffers from outliers [16] and has limited predictive ability in the presence of nonlinear SARs in training and/or test sets [3]. To address the outlier problem, a penalty on model weights can be introduced. This requires optimizing the penalized residual sum of squares defined as:

(Eq. 7) $$min_{w}{\left|\left|X_{w}-y\right|\right|}^{2}\;+\;\alpha {\left|\left|w\right|\right|}^{2}$$

Here, *X_w_* is the estimated target value, *y* the true target value, *w* the weighting coefficient and *α* the regularization parameter determining regularization strength. This regularized least squares LR approach is generally referred to as ridge regression (RR) [17,18], which was applied herein as an advanced LR technique.

### Support vector regression

Support vector machine (SVM) [16,19] is a supervised machine learning algorithm that is widely used in chemical informatics [3]. SVM was originally introduced as a method for binary object classification (class label prediction) and ranking. The SVM algorithm aims to separate positive and negative training instances in a given feature space via a hyperplane having the largest possible margin [19]. If linear separation is not possible in a given feature space, kernel functions are applied to project the training data into higher dimensional feature spaces where linear separation might become possible [19].

Support vector regression (SVR) [20] is a variant of the SVM approach. Instead of optimizing a separating hyperplane for classification, a regression function is derived for predicting numerical values:

(Eq. 8) $$f(x)\;=\;{\text{Σ}}_{i}(\alpha -\alpha_{i^{*}})K(x_{i},x)\;+\;b$$

Here, *α* and α_i^*^_ are support vectors representing the vector *w* derived from a convex optimization procedure, *K*(*x_i_, x*) is the kernel function applied to the input feature vectors and *b* the bias parameter derived from the convex optimization procedure [20,21]. SVR is capable of fitting a LR function for nonlinear SARs by increasing the feature space dimensionality. Therefore, it has become a method of choice for nonlinear QSAR modeling and potency prediction [3]. Critical parameters during model building include the regularization term *C* and the ϵ-*insensitive tube* [20,21]. The *ϵ* parameter determines the maximally permitted prediction error during training and regularization term determines the trade-off between model complexity and error penalization.

### Free-Wilson formalism

**Figure 1.**
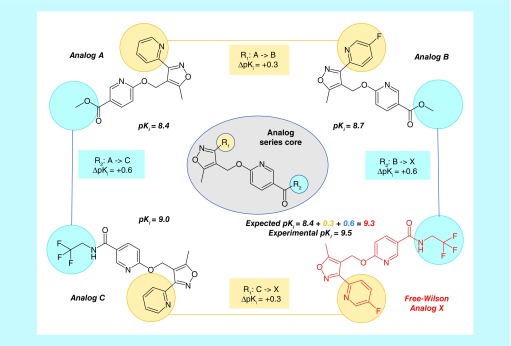
Principles of Free-Wilson analysis. Shown are four analogs from the same AS that are active against GABA receptor alpha-5 subunit (AS 5; ChEMBL target ID 5112). For each compound, its logarithmic experimental potency (pK_i_) value is reported. In addition, the core structure of the AS is depicted in the center and the two substitution sites R_1_ and R_2_ are highlighted in yellow and blue, respectively. Corresponding substituents in analogs are colored accordingly. Individual potency contributions of directed substitutions are reported as ΔpK_i_ values. The figure illustrates the principles of Free-Wilson predictions of compound potency. AS: Analog series.

Free-Wilson (FW) analysis is based upon the premise that chemical modifications in series of compounds are independent of each other and that associated potency changes are additive [22,23]. The additivity assumption represents an approximation because there might also be cooperativity between substitution sites. However, in practice, the FW approximation often holds, providing a basis for meaningful compound potency predictions [23]. Principles of FW analysis are illustrated in Figure 1. Exemplary EAs with activity against the GABA receptor alpha-5 subunit are shown and their experimentally measured logarithmic K_i_ values are given. Structural relationships between EAs were established by searching for MMPs. In this example, analog A forms distinct MMP relationships with analog B and C as a consequence of structural modifications at the first and second substitution site (R_1_ and R_2_), respectively. By contrast, analogs B and C do not form an MMP because they differ at both substitution sites. Analogs A and B share the same substituent at R_2_ while the pyridine ring at R_1_ in A is fluorinated in B, which results in a potency increase ΔpK_i_ = +0.3. Conversely, analogs A and C share the same substituent at R_1_ while the methyl ester function at R_2_ in A is replaced by a tri-fluoro ethyl amide in C. This modification results in a potency increase of ΔpK_i_ = +0.6 for analog C. Combining the structural modifications that convert analog A to B and A to C, respectively, results in a new analog X. This analog forms an MMP with B and C, respectively, but not with analog A that differs from X at two sites. Following FW principles, the potency of analog X can be predicted on the basis of analog A by adding the potency changes accompanying the conversions of A to B and C, respectively, as illustrated in Figure 1. Accordingly, the pK_i_ value predicted for analog X is 9.3 (i.e., 8.4 + 0.3 + 0.6). A, B and C form the Free-Wilson neighborhood (FW NBH) of analog X. For a given FW target compound (such as X), multiple qualifying FW NBHs may exist. In this case, potency predictions are typically averaged over all qualifying FW NBHs.

For this FW prediction example, analog X was ‘virtualized’ (i.e., considered as a VA) since it also belonged to the AS with activity against the GABA receptor alpha-5 subunit. The predicted value of pK_i_ = 9.3 was only slightly lower than the experimentally observed potency of pK_i_ = 9.5, illustrating the utility of FW predictions when the additivity approximation applies.

### Generation of FW analogs

To complement COMO-derived VA populations a new algorithm was implemented to generate VAs suitable for FW analysis (termed FW VAs). The FW VA algorithm consists of the following steps:

Given an AS core structure with indexed substitution sites, all EAs and their site-specific substituents are collected.
For EAs, all possible MMPs are generated and organized in a MMP network (using the Python Networkx package [24]) where nodes represent EAs and edges pairwise MMP relationships.
For each MMP, exchanged and conserved substituents are stored and assigned to the MMP edge in the network.
Exhaustive search for FW NBHs (according to Figure 1) is performed and detected FW NBHs are stored.
For each FW NBH, the direction of the MMP-defining substituent exchanges is determined according to Figure 1 (i.e., A to B, A to C) and the corresponding newly introduced substituents are recorded.
For each FW NBH, new substituents are added to the AS core (using the OpenEye toolkit [25]) generating a new FW VA for the NBH.
Unique FW VAs associated with one or more FW NBHs are retained.
FW NBHs define existing FW analogs (FW EAs) that are also sampled.

For each AS, varying numbers of FW NBHs, FW EAs and FW VAs were obtained, depending on the underlying MMP distribution. The calculations identified FW EAs for further analysis and generated FW VAs for potency prediction and candidate selection.

### Potency predictions

#### Regression models

For each AS, QSAR models using RR and SVR were independently generated via double (internal and external) cross-validation [26] using scikit-learn [27]. For each analog, the extended connectivity fingerprint with bond diameter 4 [28] was calculated and folded into a 1024-bit feature vector using RDKit [29] as a molecular representation. Initially, each AS was randomly partitioned into training/test data (80%) and external validation sets (20%) 35 times to ensure statistically sound model evaluation. In addition, it was monitored that the potency of each FW EA from a given series was externally predicted at least once using RR and SVR. Training and test data were subjected to fivefold internal cross-validation. During internal cross-validation optimal hyper-parameters were selected for each model. These hyper-parameters were subsequently used for prediction of the external validation set for the same independent trial. For SVR, a parameter grid of 18 *C* and 5 *ϵ* values was optimized in combination with the Tanimoto kernel [30]. For RR, seven different *α* values were tested during hyper-parameter optimization. The RR and SVR models were also used for predicting the potency of FW VAs for each AS. Predictions were averaged over all models.

#### FW predictions

For each AS, compounds forming FW NBHs were identified. Each participating FW EA was virtualized and its potency was predicted as the mean over all FW NBHs in which it occurred. Analogous predictions were carried out for newly generated AS-specific FW VAs.

#### Model evaluation

The performance of QSAR models can be evaluated using different statistical measures [3]. Herein, the coefficient of determination termed R^2^ was used as the most popular measure, which is defined as:

(Eq. 9) $$R^{2}\;=\;1-\frac{{\text{Σ}}_{i}{\left(y_{i}-f_{i}\right)}^{2}}{{\text{Σ}}_{i}{\left(y_{i}-\bar{y}\right)}^{2}}$$

Here, *y_i_* is the true value of instance *i*, *f_i_* the predicted value of instance *i*, and $\bar{y}$ the mean of all true test instance values. The numerator represents the residual sum of squares and the denominator is the total sum of squares. The maximal value of R^2^ is 1, which results from perfect correlation between predicted and true values. A value of 0 (or negative value) for R^2^ means that the performance of a model is equal to (or worse than) simple value averaging.

## Results & discussion

### Study goal

COMO was originally designed as a diagnostic approach to aid in the evaluation of LO efforts by combining quantitative assessments of chemical saturation and SAR progression. Chemical saturation analysis utilizes AS-dependent VA populations to chart chemical space around an AS. Such VAs might thus be assigned a dual purpose as diagnostic chemical entities and as potential candidates for AS expansion. This dual role provides the opportunity to generate a unique computational approach that combines LO diagnostics with compound design and candidate prediction. The corresponding workflow includes the analysis of optimization characteristics of ASs, identification of series with further development potential and use of predictive models to screen AS-specific VA populations for preferred candidate compounds. Extending COMO for combined diagnostic AS analysis and prospective series expansion was the major goal of our study.

### Diagnostic scoring

**Figure 2.**
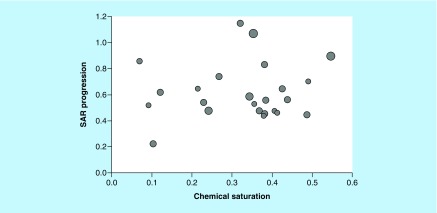
Compound optimization monitor diagnostic scores. The scatter plot compares COMO chemical saturation **(S)** and SAR progression **(P)** scores for ASs from ChEMBL (version 25). Each dot represents a series and is scaled in size according to the number of analogs. Different combinations of S and P scores distinguish ASs at different LO stages. AS: Analog series; COMO: Compound optimization monitor; LO: Lead optimization; SAR: Structure–activity relationship.

For our analysis, new ASs were assembled that contained at least 100 compounds. As reported in Table 1, the majority of these ASs consisted of 100–200 analogs. The three largest ASs comprised 237, 259 and 264 compounds, respectively. Hence, newly identified ASs were of considerable size. Initially, it was investigated if these ASs displayed different characteristics suitable for our analysis. Therefore, COMO scores were calculated. Figure 2 compares S and P scores for the ASs. COMO scoring clearly distinguished between ASs, revealing different degrees of chemical saturation and SAR progression that did not correlate with AS size. None of the ASs displayed a combination of high chemical saturation and low SAR progression, which would represent a termination criterion [9]. Hence, all ASs were still expandable through the generation of new analogs and were thus suitable for our analysis.

### Regression QSAR models

**Figure 3.**
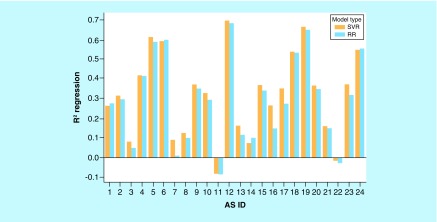
Performance of regression models. On the vertical axis, mean coefficients of determination (R^2^) for regression models are reported. The horizontal axis lists ASs using their IDs according to Table 1. For each AS, two R^2^ values are given for RR (blue) and SVR (orange) models. AS: Analog series; ID: Identification; RR: Ridge regression; SVR: Support vector regression.

A prerequisite for meaningful screening of AS-specific VA populations is the derivation of accurate QSAR models for given ASs. Accurate models make it possible to carry out meaningful predictions for VAs and prioritize candidate compounds for further exploration. Therefore, we generated standard RR and SVR models for all 24 ASs and evaluated their predictive performance. In our study, no decision tree methods were considered, given that SVR is a widely applied standard in the QSAR field. The results are shown in Figure 3 and reveal that model performance was highly heterogeneous, depending on the AS. For the majority of ASs, no predictive regression models were obtained. Models with R^2^ values exceeding 0.6 were only observed in a few cases. Overall, there was a slight increase in prediction accuracy for the more complex SVR over the simple RR models. Limited prediction accuracy of regression models for ASs is frequently observed if models are not iteratively fine-tuned for individual series. However, for systematic AS expansion, robust predictive models with meaningful accuracy are required. Clearly, on the basis of our test calculations, limited accuracy of standard regression models prohibited their general use for our purposes. Therefore, alternative predictive approaches were explored.

### FW predictions

**Figure 4.**
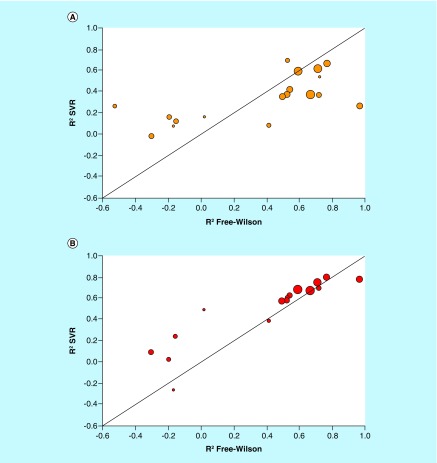
Comparison of Free-Wilson and support vector regression predictions. Scatter plots compare R^2^ values of FW and SVR predictions for individual ASs. Each dot represents an AS that is scaled in size according to the number of FW EAs. The diagonal corresponds to perfect correlation between calculated coefficients. **(A)** FW versus global SVR predictions (according to Figure 3). **(B)** FW versus SVR predictions on FW EA subsets. AS: Analog series; EA: Existing analog; FW: Free-Wilson; SVR: Support vector regression.

We reasoned that FW-type predictions following the formalism illustrated in Figure 1 might provide an alternative. This assumption was based on the local nature of FW predictions involving separate NBHs. Locally confined predictions would alleviate the need for building regression models of entire ASs that might be affected by the presence of SAR discontinuity. Therefore, we systematically searched the 24 ASs for FW EAs enabling local predictions. Varying numbers of up to 162 FW EAs were detected in 19 ASs (Table 1). For 18 of these ASs (one with only four FW EAs was excluded), systematic FW predictions were carried out. For this purpose, each FW EA was virtualized once in each NBH it occurred. Figure 4A shows R^2^ values for FW and global SVR predictions. Compared with regression modeling, the results were much more promising. In this case, 11 of the 18 qualifying ASs yielded FW predictions with R^2^ values in the range of >0.5 to 1.0 (>0.6 for seven ASs). Predictions on the seven remaining ASs with typically only small numbers of FW EAs and NBHs essentially failed. As a control, potency predictions for FW EAs using SVR models were extracted from all external validation sets and separately evaluated, as shown in Figure 4B. Surprisingly, for the 11 ASs with promising FW predictions, the potency of FW EAs was also predicted with higher accuracy using SVR models than other external validation instances. These predictions were comparable with FW analysis. Spearman correlation coefficients of potency values predicted by FW analysis and SVR models were high, ranging from 0.82 to 0.98. These improvements might be attributable to nearest neighbor effects among FW EAs.

**Figure 5.**
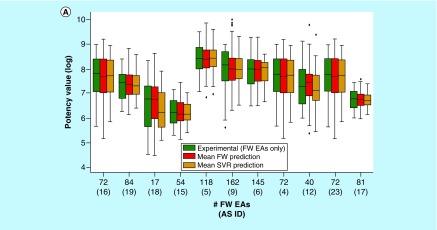

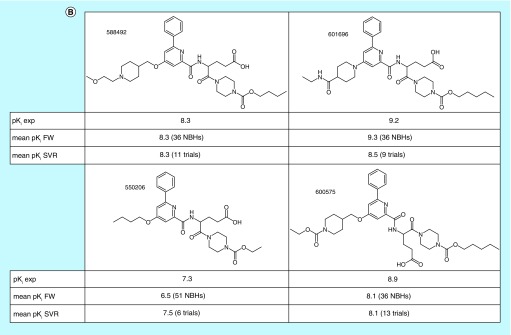
Experimental and predicted potency values. **(A)** Box plots compare experimental and predicted potency value distributions. Each triplet represents one of 11 ASs yielding predictive FW and SVR models. The y-axis reports log. potency values and the x-axis the number of FW EAs. Numbers in parentheses are AS IDs. Distributions of experimental potency values (green), mean FW predictions (red) and mean SVR predictions (orange) are reported for FW EAs. **(B)** Individual predictions are shown for four exemplary FW EAs (with ChEMBL IDs) from the same AS with activity against purinergic receptor P2Y12 (AS 6; ChEMBL target ID 2001). In the table inserts, the first row contains the experimental potency values of each analog and the second row the mean FW-predicted potency values (with the corresponding number of FW NBHs in parentheses). The third row contains the mean SVR-predicted potency values (with the corresponding number of individual prediction trials in parentheses). AS: Analog series; EA: Existing analog; FW: Free-Wilson; ID: Identification; NBH: Neighborhood; SVR: Support vector regression.

For the 11 AS, we also compared the experimental potency distribution of FW EAs with predicted distributions, as shown in Figure 5A. In the majority of cases, similar distributions and median values were observed. Notable differences between experimental and predicted potency distributions were only detected for three ASs. Figure 5B shows four exemplary FW EAs for which FW potency predictions over 36 to 51 NBHs and SVR predictions over six to 11 trials were available. These examples represented different levels of prediction accuracy. For one compound (top left), the experimental potency was exactly predicted by both FW and SVR. For another (bottom right), both methods under-predicted the experimental value by 0.8 log units. For the remaining two examples, one of the two approaches was slightly more accurate than the other. However, in all cases, the experimental potency was predicted well within an order of magnitude using both FW and SVR. Such predictions are meaningful taking experimental accuracy limits into consideration.

Thus, taken together, the results indicated that predictions of FW EAs focusing on local NBHs were much more promising than results obtained with regression models for entire ASs. Therefore, preference was assigned to FW analysis for compound potency predictions. Ultimately, such predictions must be carried out on VAs in order to prioritize candidate compounds. Therefore, in the next step, COMO VA populations were further analyzed.

### FW VAs

The diagnostic VA populations generated for the 24 ASs and used to calculate the COMO scores in Figure 2 were screened for VAs that complemented FW NBHs. These FW VAs qualified for FW predictions. However, diagnostic VA populations only contained few if any FW VAs. This was a likely consequence of using a large pool of diverse substituents for VA enumeration (see Methods). Therefore, to make FW analysis a practical option for potency prediction, we complemented diagnostic VA populations with new FW VAs. These VAs were specifically designed to complete FW NBHs in given ASs. Therefore, we implemented a new algorithm to generate FW VAs on the basis of EAs, as detailed in the Methods section. Application of this algorithm yielded between 100 and 5798 FW VAs for all but one of the 24 ASs, as reported in Table 1. Each AS contained multiple substitution sites with 45–265 available substituents that were recombined for FW analog generation. Accordingly, in some cases, large VA ensembles with several thousand compounds were obtained. Hence, through complementary analog design, COMO’s VA populations were significantly enriched with FW VAs as potential candidate compounds for AS expansion.

### Pilot predictions

To further evaluate the general suitability of FW VAs for AS expansion, potency predictions were carried out using both FW analysis and SVR models for the 11 ASs for which predictions of FW EAs succeeded. The underlying idea was that FW VA ensembles should contain FW VAs having higher predicted potency than EAs. Such FW VAs would represent preferred candidates for experimental evaluation.

**Figure 6.**
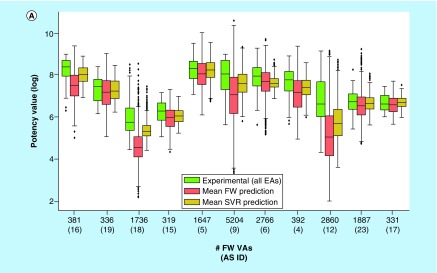

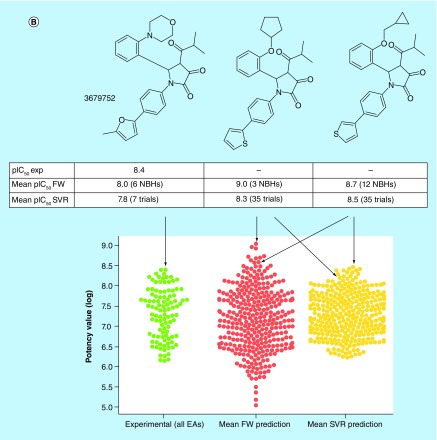
Potency predictions for Free-Wilson virtual analogs. **(A)** Box plots compare experimental potency value distributions of 11 ASs according to Figure 5A with potency predictions of corresponding FW VA populations. The y-axis reports logarithmic potency values and the x-axis the number of FW VAs per series. Numbers in parentheses are AS IDs. The experimental potency distribution of all EAs per series is displayed in light green, the FW-predicted VA potency distribution in red and the corresponding SVR-predicted distribution in orange. **(B)** Exemplary VAs (middle and right) are shown that were predicted to have higher potency than the most potent EA (left) of an AS active against the P2X purinoceptor 3 (AS 19; ChEMBL target ID 2998). In beeswarm plots below (color-coded according to the box plots), the exemplary compounds are indicated using arrows. AS: Analog series; EA: Existing analog; FW: Free-Wilson; ID: Identification; NBH: Neighborhood; SVR: Support vector regression; VA: Virtual analog.

Figure 6A compares the experimental potency value distribution of the 11 ASs with potency value distributions predicted for FW VAs using FW analysis and SVR. Predicted potency value distributions were generally lower than experimental distributions. In all but one case, the predicted median potency was lower than the experimental median. This was principally meaningful because FW VA ensembles should also contain a variety of inactive analogs. Consistent with this expectation, FW analysis predicted a number of FW VAs from different ASs to be inactive. However, the potency value distributions predicted by FW analysis typically covered a wide range. For each AS, at least a few FW VAs were consistently predicted by FW analysis to be more potent than the most potent EAs. This was an encouraging observation, providing a basis for FW VA prioritization in practical applications.

SVR-predicted distributions were generally narrower than FW distributions. Different from FW analysis, SVR is intrinsically limited to interpolative potency predictions falling within the range of training data. Thus, the potency of a few FW VAs that were predicted by SVR to be more potent than experimental analogs fell within the range of the permitted absolute prediction error of the models. Consequently, for only three ASs, potencies beyond the highest experimental value were observed.

Figure 6B shows the most potent compound from an AS representing a FW EA whose logarithmic potency value (pIC_50_ = 8.4) was well predicted using both FW analysis (pIC_50_ = 8.0) and SVR (pIC_50_ = 7.8). In addition, two FW VAs of this compound are depicted that were predicted to be most potent by FW analysis (pIC_50_ = 9.0) and SVR (pIC_50_ = 8.5), respectively. These two FW VAs were only distinguished by a cyclopentyl ether to methyl cyclopropanyl ether substitution. Both analogs were predicted by FW analysis to be more potent than the FW EA.

Taken together, the result of pilot predictions on newly generated FW VAs indicated that candidates for AS expansion could be consistently selected on the basis of FW analysis. While the activity state of preferred FW VAs remains unknown prior to experimental evaluation, the calculations revealed potential candidates. Their prioritization was further supported by meaningful predictions of FW EA potency.

It is important to note that the generation of FW VAs does not yield novel substituents because the substituents are sampled from existing compounds. Instead, novel core-substituent combinations are obtained. By design, FW VAs are enumerated to enable frequent FW predictions.

### FW centric saturation diagnostic

The newly introduced FW VA algorithm made it also possible to further extend COMO’s diagnostic scoring scheme by focusing on the saturation of FW NBHs. This additional scoring opportunity provided a close link between NBH characteristics and prospective design.

The number of FW EAs and FW VAs per AS depends on pairwise relationships between EAs captured by MMPs. Increasing numbers of FW NBHs per FW EA support reliable potency predictions. To quantitatively assess these distributions, we introduce an additional FW NBH saturation score N, which quantifies the saturation of an AS with FW NBHs:

(Eq. 10) $$N\;=\;1-\frac{n_{Fw\;EA}}{n_{Fw\;NBH}}$$

**Figure 7.**
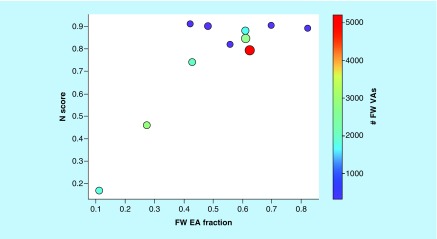
Free-Wilson neighborhood saturation scores. N scores are shown for ASs yielding predictive models as a function of increasing FW EA fraction, defined as the proportion of FW EAs among all EAs. Dots represent ASs that are scaled in size by the number of analogs per series and color-coded according to the number of algorithmically generated FW VAs per series. AS: Analog series; EA: Existing analog; FW: Free-Wilson; VA: Virtual analog.

Accordingly, increasing N values result from increasing numbers of FW NBHs per FW EA. In addition to AS size, this also increases the statistical likelihood to identify FW VAs. Large N scores indicate the presence of NBH behavior among EAs and the potential to further expand ASs with prioritized and correctly predicted FW VAs. Figure 7 reveals that N scores of ASs typically increased with increasing proportions of FW EAs among EAs. Accordingly, this measure of NBH content was a meaningful addition to COMO’s scoring repertoire. Furthermore, nearly all ASs for which well-performing predictive models were obtained produced high N scores indicating reliable potency predictions. Such predictions can only be obtained in the presence of SAR continuity, which also provides a basis for optimization of other properties during later stages of LO. This is the case because in the presence of SAR continuity, substitutions will lead to moderate changes in potency, making it possible to balance multiple properties.

## Conclusion

In this work, the diagnostic COMO approach was used as a platform to develop the first computational methodology for combining the assessment of progress in LO and expansion of ASs. To these ends, complementary strategies for analog design and potency prediction were explored. FW analysis was found to be a preferred approach for potency prediction across different ASs. Given its local nature, interpretability, and low computational complexity, FW analysis was attractive from several points of view. However, to enable extensive FW predictions for candidate prioritization, VA populations needed to be enriched with FW VAs. This was accomplished by developing a new dual-purpose algorithm to search for FW EAs and generate AS-specific FW VAs as source for candidate compounds. For FW VAs, FW analysis yielded predictions covering a wide potency range, including FW VAs predicted to be more potent than EAs. Algorithmic generation of FW NBH also led to the introduction of a new NBH-based saturation score. This score is applicable to estimate the likelihood of obtaining FW VAs and FW predictions over multiple NBHs. Taken together, our results indicate that computational LO diagnostics, analog design and candidate prioritization can be effectively integrated.

## Future perspective

Combining LO diagnostics with analog design has significant potential for practical applications. ASs can be profiled on a large scale and series with strong potential for further development can be selected. In addition, parallel series can be monitored for chemical saturation and SAR progression characteristics during late stages of LO and close-in VAs can be generated. COMO offers new opportunities to closely link AS evaluation and expansion. Assessment of chemical saturation and SAR progression has been extended by FW NBH centric scoring to identify ASs that have potential for further expansion through FW analysis. For evolving series, FW EAs can be identified using our new algorithm and then systematically predicted to assess potency prediction accuracy. For qualifying ASs, the dual-purpose algorithm can be applied to generate FW VAs. The resulting FW VA ensembles provide the basis for a second round of potency predictions to prioritize candidates for synthesis. As we have shown, SVR models also yield consistently more accurate potency predictions for FW EAs than other EAs. Hence, SVR also merits consideration for prediction of FW VAs. FW analysis was shown to produce predictions covering wide potency ranges, typically including candidates with higher predicted potency than EAs. By contrast, potency ranges predicted using SVR were smaller. However, for potency predictions of FW VAs, both FW analysis and regression modeling might best be applied in parallel to determine if most potent FW VA candidates from a given ensemble are consistently predicted. The combined diagnostic and compound design approach can be practically applied to ASs of any source. Future refinements and extensions of the methodology will have several focal points. A major limiting factor for analog prioritization is the dependence of potency prediction accuracy on the nature of ASs, which represents a general problem in the QSAR field. Hence, for an attractive AS, it might not be possible to generate reasonable predictive models to guide analog design. Accordingly, it will be beneficial to further explore and characterize SAR features that limit prediction accuracy. Any potential progress in the area is highly desirable. Furthermore, specifically for our methodology, an area of high priority for future development will be the extension of diagnostics and predictions to other LO-relevant molecular properties. Such development efforts are currently hindered by limited availability of high-quality data beyond potency measurements in the public domain. It is hoped, however, that such data will become increasingly available in the near future, in particular, through academic drug discovery efforts and/or increasing collaborations between the pharmaceutical industry and academia.

**Executive summary**

- Lead optimization (LO) plays a central role in medicinal chemistry but is vulnerable.
- Computational approaches providing decision support are rare.
- The compound optimization monitor (COMO) method quantitatively assesses optimization progress.
- LO diagnostics and compound design have not yet been combined.

**Methods**

- Principles of COMO diagnostics are summarized.
- Key scores are explained.
- The identification of analog series is described.
- Different methods for compound potency prediction are compared.
- A new algorithm for FW (Free-Wilson)-oriented analog design is introduced.
- Model building and test calculations are detailed.

**Results & discussion**

- Study rationale and goals are emphasized.
- Alternative predictive models are evaluated and compared.
- Complementary virtual analog (VA) ensembles for FW analysis are generated.
- FW predictions of candidate compounds are explored.
- A new FW neighborhood centric COMO score is introduced.

**Future perspective**

- A workflow for practical applications of the extended COMO approach is provided.
- Areas for future development are highlighted.
- Extending the approach to multiple LO-relevant properties is a priority.
